# Recent Progress in the Regioselective Biotransformation and Multitarget Therapeutic Potential of Ginsenoside Rd

**DOI:** 10.3390/molecules31071201

**Published:** 2026-04-04

**Authors:** Xingang Shen, Kun Ming, Hongjiao Shi, Jiawei Li, Ye Yang, Wenping Zhang, Xiuming Cui, Xiaoyan Yang

**Affiliations:** Faculty of Life Science and Technology, Kunming University of Science and Technology, Kunming 650500, China; dashen@stu.kust.edu.cn (X.S.); 20232218048@kust.edu.cn (K.M.); shihongjiao@stu.kust.edu.cn (H.S.); 20252118101@stu.kust.edu.cn (J.L.); yangye@kust.edu.cn (Y.Y.); wpzhang@kust.edu.cn (W.Z.); 20120094@kust.edu.cn (X.C.)

**Keywords:** ginsenoside Rd, regioselective biotransformation, neuroprotection, oncology, hepatoprotection

## Abstract

Ginsenoside Rd, a protopanaxadiol (PPD)-type tetracyclic triterpenoid saponin, has emerged as a promising bioactive constituent for multitarget therapeutic interventions. However, its natural abundance in the source plant is extremely low, making direct extraction both costly and inefficient. This review systematically summarizes the latest research progress on regioselective biotransformation strategies for Rd production since 2022. Furthermore, it comprehensively reviews recent advances in the diverse pharmacological activities of Rd. Beyond its well-recognized neuroprotective effects against neurological disorders including Alzheimer’s disease and Parkinson’s disease, we also highlight its antitumor activity and multitarget protective effects in liver diseases. This review provides a theoretical basis for developing Rd as a high-value nutraceutical and therapeutic candidate for systemic health.

## 1. Introduction

Ginsenoside Rd (Rd) is predominantly found in plants of the Panax genus, such as *Panax ginseng* (Renshen), *Panax notoginseng* (Sanqi), and *Panax quinquefolius* (American ginseng) [1,2,3]. Its natural abundance is generally low, making large-scale extraction directly from plant materials difficult [4]. Studies have shown that many highly abundant protopanaxadiol (PPD)-type saponins in *Panax* species—such as the dammarane-type tetracyclic triterpenes Ginsenosides Rb_1_, Rb_2_, Rb_3_ and Rc—share a similar chemical structure with Rd. They all possess a disaccharide chain linked at the C-3 position, but differ in the sugar chains or glycosyl groups attached at the C-20 position [5]. Following oral administration, these saponins undergo biotransformation, in which specific glycosyl groups are hydrolyzed by digestive enzymes or gut microbiota, leading to their conversion into Rd. This biotransformation process provides a theoretical foundation for the efficient preparation of Rd.

Neurological disorders, including Alzheimer’s disease, Parkinson’s disease, epilepsy, and ischemic stroke, exhibit complex pathological mechanisms, often involving multiple processes such as neuroinflammation, neuronal apoptosis, and neuronal excitotoxicity [6,7,8]. In recent years, Rd has garnered widespread attention for its modulatory effects on the nervous system, owing to its good efficacy and favorable safety profile [9]. It has demonstrated immense potential and promising application prospects in the treatment of neurological diseases [10].

This review systematically synthesizes research breakthroughs since 2022, focusing on the regioselective biotransformation and the expanding pharmacological landscape of Rd. To ensure methodological transparency, a systematic literature search was conducted via PubMed, Web of Science, and Google Scholar using keywords such as ‘Ginsenoside Rd’, ‘Biotransformation’, and ‘Pharmacological effects’. By integrating the latest evidence on its anti-tumor activities, hepatoprotective mechanisms, and established neuroprotective roles, this work provides a theoretical basis for developing Rd as a potential multitarget therapeutic candidate.

## 2. Latest Research Progress on the Biotransformation Pathways of Rd

Compared to traditional chemical synthesis, biotransformation offers several advantages, such as high selectivity, mild reaction conditions, and environmental sustainability. Conversely, it has progressively become a common method for the preparation of rare Rd [11,12]. Reviews by Li et al. [13] and Song et al. [14] have systematically summarized the relevant research prior to 2022. This review primarily focuses on new discoveries post-2022, elucidating the latest breakthroughs and developmental trends in the production of Rd via ginsenoside biotransformation.

### 2.1. Conversion of Ginsenoside Rb_1_ to Rd

**Figure 1:**
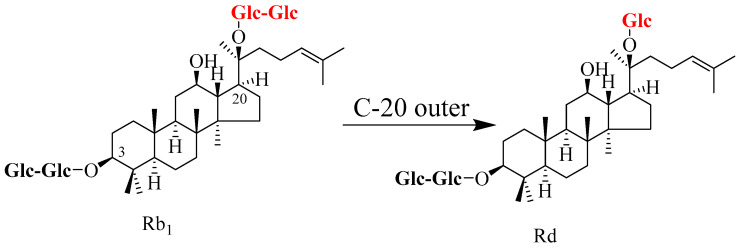
Schematic representation of the regioselective biotransformation pathway from ginsenoside Rb_1_ to Rd. The process primarily involves the specific hydrolytic cleavage of the glycosidic bond at the C-20 position. Abbreviations: Rb_1_, ginsenoside Rb_1_; Rd, ginsenoside Rd.

Ginsenoside Rb_1_ is one of the most abundant components among PPD-type ginsenosides. Its molecular structure is characterized by two glucosyl groups linked at the C-3 position via a *β*-1,2-glycosidic bond and a gentiobiosyl group linked at the C-20 position via a *β*-1,6-glycosidic bond [15]. Therefore, the conversion of Ginsenoside Rb_1_ to Rd is the most common and most extensively studied pathway [16]. This process involves the hydrolysis of the terminal (outer) *β*-D-glucosyl group at the C-20 position of the Rb_1_ molecule, which yields Rd (Figure 1). Microbial transformation and enzymatic transformation are the two principal pathways for the conversion of Rb_1_ to Rd [17]. In recent years, researchers have made significant progress in screening highly efficient transforming microbial strains and identifying novel conversion enzymes (Table 1).

#### 2.1.1. Microbial Transformation

Bacteria play an important role in microbial transformation [38]. Yi et al. [29] reported that *Levilactobacillus brevis* THK-D437, isolated from the traditional Korean fermented food kimchi, could efficiently convert Rb_1_ to Rd. Using ginsenoside Rb_1_ as the substrate, the Rd content increased by 295.8% compared with the pre-fermentation level. Nguyen et al. [20] co-fermented *L. brevis* QD-1 with Ngoc Linh ginseng and found that Rd was the main component in the fermentation product, with its content increasing significantly after 48 h of fermentation.

Furthermore, fungi also play a significant role in microbial transformation. Niu et al. isolated *Fusarium proliferatum* G11-7 from *Cajanus cajan* and co-cultured this strain with *P. notoginseng*. The experimental results showed that the Rd content increased 3.67-fold compared to the untreated control, while the Rb_1_ content simultaneously decreased. This study indicated that the endophytic fungus G11-7 can significantly elevate the content of Rd by transforming Rb_1_ [34]. Zhao et al. employed a bidirectional solid-state fermentation technique, co-culturing *Cordyceps militaris* with *P. quinquefolius*. They found that the ginsenoside Rb_1_ content decreased as fermentation progressed, whereas the Rd content increased from an initial 14.46 mg to 134.35 mg [18]. Similarly, Gao et al. utilized the culture medium of *Irpex lacteus* to biotransform ginsenoside Rb_1_. The results revealed that Rb_1_ was converted to Rd via deglycosylation at the C-20 position [31].

#### 2.1.2. Enzymatic Transformation

*β*-glucosidases are commonly utilized for the transformation of ginsenoside Rb_1_ and are considered to possess good conversion activity. Yu et al., while investigating the catalysis of ginsenoside Rb_1_ into the rare saponin F_2_ using a *β*-glucanase derived from *Aspergillus niger*, discovered that at a temperature of 50 °C and a pH of 3.4, the *β*-glucosidase specifically catalyzed the hydrolysis of the sugar chain at the C-20 position of ginsenoside Rb_1_, yielding Rd. Furthermore, this reaction could be completed within 12 h [19]. Liu et al. isolated a novel *α*-amylase (StAMY) from *Streptococcus thermophilus* 17140. Using ginsenoside Rb_1_ as the substrate, they reacted it with the purified recombinant StAMY under conditions of 50 °C and pH 5.5. It was observed that the ginsenoside Rb_1_ content decreased post-reaction, and Rd was detected in the reaction products. This study demonstrated that StAMY can degrade ginsenoside Rb_1_ into Rd, and possesses the ability to hydrolyze the *β*-glycosidic bonds of ginsenosides [21].

### 2.2. Conversion of Ginsenoside Rb_2_ to Rd

**Figure 2:**
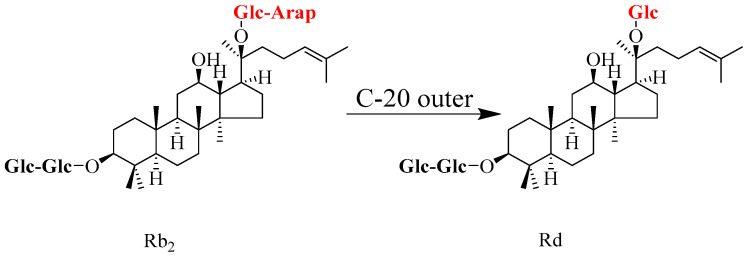
Schematic representation of the regioselective biotransformation pathway from ginsenoside Rb_2_ to Rd. The process specifically involves the hydrolytic cleavage of the terminal arabinose moiety at the C-20 position. Abbreviations: Rb_2_, ginsenoside Rb_2_; Rd, ginsenoside Rd.

In addition to the aforementioned conversion of ginsenoside Rb_1_ to Rd, the pathway for the conversion of ginsenoside Rb_2_ to Rd has also received sustained attention. Recent related studies are summarized in Table 2. Ginsenoside Rb_2_ is structurally similar to Rb_1_, but its outer glycosyl group at the C-20 position is an *α*-1,6-linked arabinofuranosyl group [39]. The precise hydrolysis of this structure can be achieved by using highly efficient and specific glycosidases or selected microorganisms, as illustrated in Figure 2.

#### 2.2.1. Microbial Transformation

Bacteria can exhibit ginsenoside Rb_2_ transformation activity. Tang et al. cultured *Bifidobacterium animalis* subsp. *lactis* CCFM1274 in GE medium and found that ginsenoside Rb_2_ was consumed [28]. Compared with the pre-fermentation level, the content of Rd increased significantly by 43%. This study demonstrates that CCFM1274 is capable of converting Rb_2_ to Rd in vitro.

#### 2.2.2. Enzymatic Transformation

Enzymes are key factors influencing biotransformation. Lu et al. isolated and identified Xyaf313 from the endophytic fungus *Chaetomium globosum* DX-THS3 [22]. This enzyme possesses the dual activities of *α*-*L-arabinofuranosidase* and *β*-D-xylosidase. Utilizing the ability of Xyaf313 to selectively hydrolyze the glycosidic bond at the C-20 position of PPD-type ginsenosides, ginsenoside Rb_2_ can be specifically converted to Rd. Furthermore, Zhou et al. isolated a novel GH1 *β*-glucosidase from *Fervidobacterium pennivorans* DSM9078 [25]. This enzyme exhibits strong thermostability and can effectively cleave the external *β*-(1→6) glycosidic bond at the C-20 position of ginsenoside Rb_2_ under conditions at 100 °C, thereby converting ginsenoside Rb_2_ to Rd. Moreover, they analyzed the interactions between the ginsenoside and amino acid residues via molecular docking, revealing the reaction mechanism of this conversion.

### 2.3. Conversion of Ginsenoside Rb_3_ to Rd

**Figure 3:**
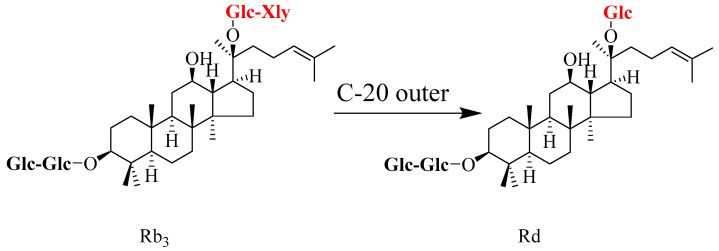
Schematic representation of the regioselective biotransformation pathway from ginsenoside Rb_3_ to Rd. The process specifically involves the cleavage of the terminal xylose moiety at the C-20 position. Abbreviations: Rb_3_, ginsenoside Rb_3_; Rd, ginsenoside Rd.

The structural characteristic of ginsenoside Rb_3_ is the presence of a *β*-D-glucosyl-(1–6)-*β*-D-xylosyl group linked at the C-20 position. This implies that achieving the conversion of Rb_3_ to Rd requires a *β*-D-xylosidase to highly and specifically hydrolyze the terminal *β*-D-xylosidic bond of the sugar chain at the C-20 position [40], as shown in Figure 3. In recent years, with the continuous discovery of *β*-xylosidases with higher specificity and activity, research into the conversion of Rb_3_ to Rd has garnered increasing attention, as summarized in Table 3.

#### 2.3.1. Microbial Transformation

Tang et al. cultured *Bifidobacterium animalis* subsp. *lactis* CCFM1274 in GE medium and observed that ginsenoside Rb_3_ was also consumed [28]. Compared with the pre-fermentation level, the content of Rd increased significantly. This study also demonstrated that CCFM1274 is capable of converting Rb_3_ to Rd in vitro.

#### 2.3.2. Enzymatic Transformation

Zhao et al. cloned a 1197 bp *β*-xylosidase gene (BaXyl5B) from *Bifidobacterium adolescentis* and expressed it in *E. coli* BL21 [41]. Using ginsenoside Rb_3_ as the substrate for biotransformation, analysis by TLC and HPLC indicated that the enzyme could hydrolyze the *β*-1,6-linked xylose at the C-20 position, thereby converting ginsenoside Rb_3_ to Rd. Through molecular docking analysis, they investigated the binding mode of ginsenoside Rb_3_ within the BaXyl5B binding pocket, further elucidating the structure-activity relationship of this biotransformation. Xu et al. utilized a *β*-glucosidase (Pxbgl) from *Petroclostridium xylanilyticum* to biotransform ginsenosides in crude extracts of *P. ginseng* roots and *P. notoginseng* leaves [30].

This study found that Pxbgl could not only convert ginsenoside Rb_3_ to Rd, but also exhibited a significantly better conversion effect in the *P. notoginseng* leaf crude extract than in the *P. ginseng* root crude extract. Zhang et al. reported for the first time the process of converting Rb_3_ to Rd using a *β*-xylosidase (Ta-XylQS) derived from *Thermoascus aurantiacus*. The study showed that after recombinant expression in *Komagataella phaffii*, this enzyme could specifically catalyze the hydrolysis of substrates bearing xylosyl residues at 60 °C and pH 3.5, thereby converting Rb_3_ to Rd [42].

### 2.4. Conversion of Ginsenoside Rc to Rd

**Figure 4:**
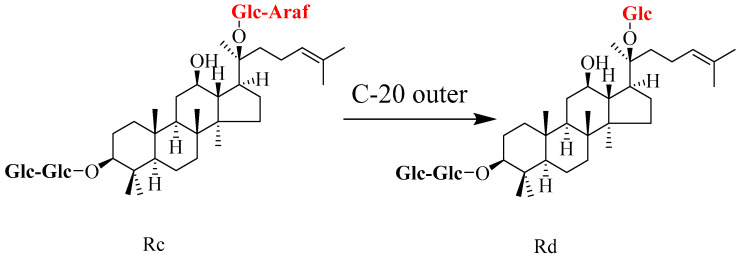
Schematic representation of the regioselective biotransformation pathway from ginsenoside Rc to Rd. The process specifically involves hydrolytic cleavage of the terminal arabinofuranose moiety at the C-20 position. Abbreviations: Rc, ginsenoside Rc; Rd, ginsenoside Rd.

In ginsenoside Rc, the outer glycosyl group at the C-20 position is an *α*-1,6-linked arabinofuranosyl-glucosyl group [43]. This structural feature means that the conversion of ginsenoside Rc to Rd requires an *α*-L-arabinofuranosidase to selectively cleave the *α*-L-arabinofuranosyl group linked at its C-20 position [44], as shown in Figure 4. Compared with Rb_1_, biotransformation studies using Rc as a substrate are relatively scarce; however, several important findings have also emerged in recent years, as summarized in Table 4.

#### 2.4.1. Microbial Transformation

In terms of microbial transformation, bacteria also exhibit Rc conversion activity. Tang et al. cultured *Bifidobacterium animalis* subsp. *lactis* CCFM1274 in GE medium and found that CCFM1274 could not only convert ginsenosides Rb_1_ and Rb_2_ to Rd in vitro, but also ginsenoside Rc [28]. Furthermore, Zhang et al. isolated a strain of *Bacillus* sp. G9y from *Panax quinquefolius*. This strain can convert ginsenoside Rc to Rd, and it was found that the generated Rd is not further transformed into other saponins [48]. This indicates that G9y can specifically convert ginsenoside Rc into the target product, Rd.

#### 2.4.2. Enzymatic Transformation

Shen et al. cloned an *α*-L-arabinofuranosidase (BpAbf51A) from *Bacillus pumilus* and heterologously expressed it in *E. coli* BL21. Their results found that, with ginsenoside Rc as the substrate, BpAbf51A could specifically remove the terminal arabinofuranosyl (Araf) residue at the C-20 position of ginsenoside Rc at 50 °C and pH 8.0, thereby realizing the biotransformation of Rc to Rd [45]. Moreover, molecular docking analysis showed that the key amino acid residues Ser213 and Asn214 bind to the arabinofuranosyl group of Rc via hydrogen bonds, with a binding energy of −7.9 kcal/mol, explaining the mechanism of its specific catalysis. Zhu et al. cloned and expressed an *α*-L-AFase from *Bacillus subtilis* for the first time. Subsequent investigation of this enzyme revealed that it can efficiently and specifically catalyze the conversion of ginsenoside Rc to the rare Rd under conditions of 40 °C and pH 5.5 [46]. In addition, Zhu et al. also cloned and expressed a novel *α*-L-arabinofuranosidase (Bsafs) from *Bacillus subtilis*. This enzyme can also specifically hydrolyze the *α*-L-arabinofuranosyl group at the C-20 position of Rc under conditions of 30 °C and pH 7.5, thereby converting Rc to Rd [47].

## 3. Pharmacological Effects of Ginsenoside Rd

### 3.1. Neuroprotective Effects of Ginsenoside Rd

Common central nervous system (CNS) diseases, including Alzheimer’s disease (AD), Parkinson’s disease (PD), ischemic stroke (IS), and epilepsy, possess complex pathogenic mechanisms (pathomechanisms) that typically involve multiple processes such as neuroinflammation, oxidative stress, neuronal apoptosis, and excitotoxicity [49,50]. Although existing therapeutic drugs can alleviate some associated symptoms to a certain extent, they are also accompanied by adverse reactions and cannot provide a definitive cure [51,52]. Consequently, identifying natural bioactive constituents from edible plants has become a major focus in the development of functional foods and nutraceuticals [53,54]. Chen et al. conducted a systematic review of the neuroprotective effects of Rd [10]. Building upon this foundation, this review synthesizes current literature to summarize the most recent research findings not covered in the previous work.

#### 3.1.1. Effect of Ginsenoside Rd on Alzheimer’s Disease (AD)

Alzheimer’s disease (AD) is a chronic neurodegenerative disorder, primarily characterized by cognitive impairments such as memory loss and executive dysfunction [55,56]. The pathomechanism of AD is highly complex and is generally considered to be associated with factors such as the hyperphosphorylation of Tau protein, deposition of *β*-amyloid (A*β*), neuronal degeneration, neuroinflammation, and mitochondrial dysfunction [57,58,59]. These pathological changes severely impact the patient’s activities of daily living, posing immense health challenges and financial burdens on patients and their families [60,61].

Aberrant cleavage of the amyloid precursor protein (APP) by *β*-secretase leads to the massive accumulation of A*β*, forming insoluble aggregates that are toxic to neurons, a key factor in the formation of AD [62]. Mi et al. predicted, using network pharmacology and molecular docking, that Rd could intervene in 311 AD-related targets, implicating multiple key pathways such as MAPK, JAK-STAT, and PI3K-Akt. They also evaluated the effect of Rd in *C. elegans* (nematodes), demonstrating that Rd significantly reduces A*β* aggregation by targeting the MAPK signaling pathway. It induces the nuclear translocation of DAF-16 to activate downstream signaling pathways, thereby counteracting A*β*-induced toxicity [63].

Acetylcholine (ACh) is one of the most important neurotransmitters in the central cholinergic system, playing a crucial role in learning and memory [64]. Acetylcholinesterase (AChE) is involved in the critical hydrolysis of ACh; therefore, inhibiting AChE has become a significant therapeutic strategy for AD [65]. Studies have found that Rd can exhibit moderate inhibitory activity against acetylcholinesterase (AChE) [66].

The hyperphosphorylation of tau protein is a primary pathological feature of AD. This pathological phenomenon leads to the formation of neurofibrillary tangles (NFTs), which disrupt microtubule stability and impair the neuronal transport system [67]. Li et al. demonstrated experimentally that Rd can modulate the activation balance of CDK5 (decreasing P25, increasing P35) by inhibiting the activity of GSK-3*β* (Tyr216). Furthermore, it can also effectively inhibit the hyperphosphorylation of tau protein at key sites such as S199/202, S396, and S404 [68].

Neuroinflammation is an inflammatory response occurring in neural tissue, which causes neural damage and leads to neurodegenerative diseases [69]. Rd can inhibit the activation of the NF-kB pathway, reducing the expression of inflammatory factors such as IL-1*β*, IL-6, IL-8, and TNF-*α*, while increasing the expression of IL-10. This leads to reduced expression of the amyloid precursor protein (APP), thereby impacting A*β* accumulation [70]. Furthermore, Wang et al. similarly found that Rd can inhibit the release of the inflammatory cytokines TNF-*α* and IL-6, activate the BDNF-mediated PI3K/AKT/CREB signaling pathway, and promote neuronal survival and synaptic plasticity [71].

#### 3.1.2. Effect of Ginsenoside Rd on Parkinson’s Disease (PD)

PD is another common neurodegenerative disorder following AD [72,73]. Its primary pathological features are the progressive loss of dopaminergic neurons in the substantia nigra pars compacta and the abnormal aggregation of *α*-synuclein [74,75]. Other factors include mitochondrial failure, oxidative stress, ferroptosis, neuroinflammation, and gut microbiota dysbiosis [76,77]. Rd possesses unique anti-inflammatory properties and can exert neuroprotective effects by enhancing mitochondrial biosynthesis [78,79,80]. Park et al., by investigating the protective effect of Rd in BV2 microglial cells, demonstrated that Rd can alleviate the microglial inflammatory response by modulating the AMPK–HDAC5 signaling pathway. Furthermore, Rd was found to inhibit the activity of HDAC5, promoting the upregulation of genes related to mitochondrial biosynthesis, thereby enhancing mitochondrial function [81]. Kim et al. extracted Rd from black ginseng and investigated its effects on the production of NO, IL-6, and TNF-*α* in LPS-induced BV2 microglial cells, finding that Rd possesses significant anti-neuroinflammatory effects [82].

After *P. ginseng* extract was fermented with *Lactococcus lactis* KC24, the Rd content increased significantly. Through the detection of neuroprotection-related pathways, the Rd-enriched fermented ginseng extract was found to have markedly improved neuroprotective effects. This phenomenon suggests the pivotal role of Rd as a major bioactive contributor to the enhanced neuroprotective activity within the complex extract [83]. These findings suggest that incorporating Rd-rich fermented products into the diet could be a viable strategy for preserving neuronal vitality and supporting long-term metabolic health during aging.

#### 3.1.3. Effect of Ginsenoside Rd on Ischemic Stroke (IS)

Ischemic stroke (IS) is a neurological disease with high morbidity and high mortality, caused by the interruption of cerebral blood flow, which leads to hypoxia in brain tissue and subsequent neuronal cell death. It is one of the main causes of death worldwide [84,85,86]. Neurological dysfunction is the primary characteristic of IS. Its pathogenesis is associated with neuroinflammation, oxidative stress, ionic imbalance, cell apoptosis, and disruption of the blood–brain barrier (BBB) [87].

Rd has been observed to alleviate brain injury caused by IS through multi-target, multi-pathway mechanisms. It exerts neuroprotective effects not only by regulating ion channels (e.g., inhibiting TRPM7 and ASIC1a expression, enhancing ASIC2a expression), but also by inhibiting PARP-1 activity, the nuclear accumulation of the NFkB p65 subunit, and reducing the release of cytochrome c and apoptosis-inducing factor, thereby inhibiting neuronal cell death and neuroinflammation [14,88,89].

Furthermore, Rd exhibits promising therapeutic potential in ischemia–reperfusion (I/R) injury. Rd exerts anti-pyroptosis effects by modulating the MicroRNA-139-5p (miR-139-5p)/forkhead box transcription factor O1 (FOXO1)/Kelch-like ECH-associated protein 1 (Keap1)/nuclear factor erythroid-2 related factor 2 (Nrf2) axis, effectively reducing the damage caused by I/R injury [90]. Pretreatment with Rd can significantly promote the recovery of neurological function after spinal cord ischemia–reperfusion injury and preserve a greater number of normal motor neurons. These findings further support the potential neuroprotective role of Rd against I/R injury [91]. Hu et al. first revealed that Rd can effectively inhibit endothelial cell ferroptosis by activating the NRG1/ErbB4 signaling pathway [92]. This subsequently triggers the activation of the downstream PI3K/Akt/mTOR pathway post-stroke, ultimately protecting blood–brain barrier permeability and thereby mitigating the effects of ischemic stroke. Moreover, Rd can also increase DAPK1 phosphorylation by inhibiting calcineurin, reduce NR2B-Ser1303 phosphorylation, attenuate NMDAR channel conductance, and ultimately counteract excitotoxicity [93].

As a natural active monomer derived from traditional Chinese medicine, research on Rd in the field of neuroprotection has achieved remarkable progress. Rd integrates multiple protective strategies—including anti-oxidation, mitochondrial support, and the promotion of neuroregeneration—offering a promising dietary strategy for supporting lifelong cerebrovascular health.

### 3.2. Anti-Tumor Effects of Ginsenoside Rd

Recent studies have demonstrated that Rd may exhibit inhibitory potential against several types of malignant cells. Its pharmacological mechanisms involve multiple dimensions, including inducing cell apoptosis, inhibiting invasion and migration, and promoting tumor cell differentiation.

In terms of inducing apoptosis and inhibiting proliferation, Wan et al. [94] reported that Rd induces apoptosis in NSCLC cells by activating the p53/Bax-mediated mitochondrial apoptotic pathway. Simultaneously, it reduces the migration and invasion of these cells by inhibiting the expression of MMP-2/MMP-9, effectively suppressing tumor cell proliferation via the intrinsic apoptotic pathway. Furthermore, Rd can downregulate the expression of the oncogenic lncRNA H19, thereby decreasing the production of its derived miR-675-5p. The reduction in miR-675-5p relieves its targeted inhibition on the tumor suppressor gene CDH1, which in turn upregulates CDH1/E-cadherin expression and ultimately inhibits the progression of tongue squamous cell carcinoma [95].

In addition to inducing cell apoptosis and inhibiting invasion and migration, Rd also possesses the potential to induce the differentiation of malignant cells into normal phenotypes. Jiang et al. [96] confirmed through both in vivo and in vitro experiments that Rd can regulate the ERK/GSK-3*β* signaling pathway, effectively inhibiting the proliferation of acute myeloid leukemia (AML) cells, prompting malignant cells to overcome differentiation barriers, inducing their transformation into mature phenotypes, and ultimately leading them toward apoptosis.

### 3.3. Pharmacological Mechanisms of Ginsenoside Rd in Liver Diseases

Mounting evidence indicates that Rd exerts multi-target protective effects in the prevention and treatment of acute liver injury, chronic liver fibrosis, and metabolic liver diseases.

#### 3.3.1. Regulation of Pyroptosis and Inflammatory Responses

Mounting evidence indicates that Rd exerts multi-target protective effects in the prevention and treatment of acute liver injury, chronic liver fibrosis, and metabolic liver diseases. Li et al. [97] discovered through network pharmacology prediction that STAT3 is one of the targets most highly associated with Rd and liver injury. They further verified in an acute liver injury model co-induced by lipopolysaccharide and D-galactosamine (LPS/D-GalN) that Rd can inhibit hepatocyte pyroptosis and the release of inflammatory factors by blocking the STAT3-NLRP3-GSDMD signaling axis. Moreover, in a thioacetamide (TAA)-induced model, Rd enhanced autophagy by activating the AMPK/mTOR/ULK1 pathway, thereby inhibiting the assembly of the NLRP3 inflammasome and alleviating inflammatory damage in liver tissue [98].

#### 3.3.2. Inhibition of Ferroptosis and Regulation of the Gut-Liver Axis

Li et al. [99] found that Rd can significantly reduce lipid peroxidation levels and effectively alleviate carbon tetrachloride-induced acute chemical liver injury by inhibiting the cGAS/STING signaling pathway. Furthermore, a study by Liu et al. [97] expanded the functional scope of Rd, revealing that Rd effectively ameliorates high-fat diet-induced metabolic dysfunction-associated fatty liver disease (MAFLD) by remodeling gut microbiota homeostasis and blocking gut-liver axis-mediated hepatic lipid peroxidation and ferroptosis.

#### 3.3.3. Alleviation of Liver Fibrosis and Extracellular Matrix Deposition

Liver fibrosis is a pathological process caused by chronic liver injury that leads to the excessive deposition of the extracellular matrix, which can progress to cirrhosis and even liver failure [100]. Cui et al. [101] revealed that Rd can significantly upregulate the expression of estrogen-related receptor α (ERRα), thereby inhibiting the P2X7 receptor (P2X7r) and its downstream inflammatory responses. In a TAA-induced fibrosis model, Rd significantly reduced the excessive deposition of the extracellular matrix (ECM), effectively delaying the progression of liver fibrosis.

#### 3.3.4. Improvement of Non-Alcoholic Fatty Liver Disease (NAFLD) and Metabolic Disorders

Non-alcoholic fatty liver disease (NAFLD) is a globally prevalent metabolic disease and a common precursor to cirrhosis and hepatocellular carcinoma [102]. Cui et al. [103] elucidated that Rd acts as a novel direct activator of SIRT6. By enhancing SIRT6 activity and upregulating downstream PPAR *α* expression, Rd can significantly promote fatty acid oxidation and inhibit oxidative stress and hepatic steatosis. This study confirmed that SIRT6 is a key molecular target for Rd in exerting its hepatic metabolic regulatory effects.

## 4. Discussion

This review systematically summarizes the latest advances in the biotransformation pathways of Rd since 2022 and provides a comprehensive overview of its diverse pharmacological effects. As a rare ginsenoside with high application value, its low natural abundance in plant sources has restricted its in-depth research and clinical translation [104,105]. However, recent studies have demonstrated that enzymatic or microbial transformation of abundant protopanaxadiol (PPD)-type saponins has become a regioselective and highly efficient strategy for Rd preparation. The application of food-grade microbial strains and specific glycosidases has laid a sustainable technological foundation for the large-scale and cost-effective production of Rd.

Beyond advances in preparation technology, the application scope of Rd has expanded considerably in recent years. Although its neuroprotective effects in neurological disorders including Alzheimer’s disease (AD), Parkinson’s disease (PD), and ischemic stroke (IS) remain a major research focus, emerging evidence indicates its promising clinical potential in antitumor therapy and hepatoprotection. Rd exerts significant antitumor activity by inducing differentiation of myeloid leukemia cells and inhibiting migration and invasion of tongue squamous cell carcinoma cells. For liver protection, Rd alleviates acute liver injury by blocking the STAT3–NLRP3–GSDMD signaling axis to suppress hepatocyte pyroptosis. Meanwhile, it improves metabolic dysfunction-associated fatty liver disease (MAFLD) by restoring gut microbiota homeostasis and activating the SIRT6/PPAR α pathway to promote fatty acid oxidation.

Mechanistically, the biological effects of Rd are not mediated by a single receptor, but achieved through holistic “multi-target, multi-pathway” interactions. In nervous system models, Rd exerts neuroprotective effects by modulating core targets including PTGS2, BCL2, and MTOR, thereby synergistically regulating multiple signaling pathways involved in inflammation, apoptosis, and cell survival. Despite these encouraging preclinical findings, important research gaps still exist. The vast majority of current evidence is derived from in vitro and animal studies, underscoring an urgent need for well-designed human clinical trials to validate its therapeutic efficacy. Future research should prioritize the translation of these molecular mechanisms into clinical practice, with particular emphasis on in-depth investigations of the long-term safety and bioavailability of Rd in diverse populations.

Notably, the studies cited in this review exhibit variations in the type of evidence presented. Some investigations utilized high-purity Rd monomers, thereby providing direct and definitive evidence of its molecular mechanisms. Conversely, other studies relied on Rd-enriched extracts. In the latter cases, while Rd is considered the primary contributor to the observed biological activities, potential synergistic effects with other saponin constituents cannot be entirely ruled out. Therefore, to ensure the precision of pharmacological conclusions, future research must further validate the findings derived from extracts through rigorous preclinical experiments using purified monomers.

## 5. Conclusions

Ginsenoside Rd is a highly promising natural bioactive compound that exhibits significant therapeutic potential in the treatment of various diseases. As summarized in this review, the utilization of biotransformation enables efficient preparation of Rd, thereby alleviating the scarcity of natural Rd to a certain extent. Beyond its well-documented neuroprotective effects against neuroinflammation and oxidative stress, emerging evidence underscores the remarkable potential of Rd in oncology and hepatoprotection. By modulating key signaling axes such as ERK/GSK-3*β*, STAT3-NLRP3-GSDMD, and SIRT6/PPAR*α*, Rd exerts a holistic “multi-target, multi-pathway” regulatory effect. However, the translation from preclinical research to clinical application remains a major challenge. Future studies should prioritize rigorous human clinical trials to validate the efficacy, long-term safety, and optimal bioavailability of Rd, thereby further unlocking its potential for applications in modern medicine and nutraceuticals.

## Figures and Tables

**Table 1:** Summary of experimental conditions and catalytic efficiencies for the bioconversion of ginsenoside Rb_1_ to Rd. This table summarizes various enzymes or microbial strains and their corresponding reaction parameters. Abbreviations: n.r., not reported.

Source	Enzyme	Conditions	Year	Ref.
*Cordyceps militaris*	*β*-glucosidase	20 °C	2025	[18]
*Aspergillus niger*	*β*-glucanase	50 °C, pH 3.4	2025	[19]
*Levilactobacillus brevis* QD-1	*β*-Glucosidase	37 °C	2025	[20]
*Streptococcus thermophilus* 17140	*α*-amylase	50 °C, pH5.5	2025	[21]
*Endophytic Chaetomium globosum*	GH43 Bifunctional Glycosidase	37 °C, pH 7.0	2025	[22]
*Aspergillus tubingensis*	*β*-glucosidase	37 °C, pH 5.0	2025	[23]
*Aspergillus tubingensis* and commercial cellulase	*β*-glucanase	55 °C, pH 4.5	2025	[24]
*Fervidobacterium pennivorans* DSM9078	GH1 *β*-glucosidase	100 °C, pH 7.5	2025	[25]
*Trichoderma reesei* mutant strain BB8	Cellulase	50 °C, pH 6.0	2025	[26]
*Penicillium fimorum*	crude enzymes	60 °C, pH 5.0	2025	[27]
*B. animalis* subsp. *lactis* SW62	n.r.	n.r.	2024	[28]
*Levilactobacillus brevis* THK-D437	*β*-glucosidase	30 °C	2024	[29]
*Petroclostridium xylanilyticum*	*β*-glycosidase	60 °C, pH 6.0	2024	[30]
*Irpex lacteus*	n.r.	28 °C	2024	[31]
*Mucor abundans*	crude enzyme	60 °C, pH 3.5	2024	[32]
*Aspergillus niger* Wu-16	mixed enzymes	55 °C, pH 4.0	2023	[33]
*Fusarium proliferatum* G11-7	*β*-glucosidase	30 °C, pH 6.0	2023	[34]
*Thermoclostridium stercorarium*	*β*-glucosidase	65 °C, pH 5.0	2023	[35]
*Lentilactobacillus buchneri* URN103L	*β*-glucosidase	35 °C, pH 5.0	2022	[36]
*Talaromyces flavus*	crude enzyme	50 °C, pH 4.5	2022	[37]

**Table 2:** Summary of experimental conditions for the bioconversion of ginsenoside Rb_2_ to Rd. This table summarizes various enzymes or microbial strains and their corresponding reaction parameters. Abbreviations: n.r., not reported.

Source	Enzyme	Conditions	Year	Ref.
*Endophytic Chaetomium globosum*	GH43 Bifunctional Glycosidase	50 °C, pH 7.0	2025	[22]
*Fervidobacterium pennivorans* DSM9078	GH1 *β*-glucosidase	100 °C, pH 7.5	2025	[25]
*Bifidobacterium animalis* subsp. *lactis* CCFM1274	n.r.	n.r.	2024	[28]
*Petroclostridium xylanilyticum*	*β*-glycosidase	60 °C, pH 6.0	2024	[30]
*Mucor abundans*	crude enzyme	60 °C, pH 3.5	2024	[32]

**Table 3:** Summary of experimental conditions for the bioconversion of ginsenoside Rb_3_ to Rd. This table summarizes various enzymes/microorganisms and corresponding reaction parameters. Abbreviations: n.r., not reported.

Source	Enzyme	Conditions	Year	Ref.
*Endophytic Chaetomium globosum*	GH43 Bifunctional Glycosidase	37 °C, pH 7	2025	[22]
*Bifidobacterium adolescentis*	*β*-D-xylosidase	30 °C, pH 6.0	2025	[41]
*Bifidobacterium animalis* subsp. *lactis* CCFM1274	n.r.	n.r.	2024	[28]
*Petroclostridium xylanilyticum*	*β*-glycosidase	60 °C, pH 6.0	2024	[30]
*Mucor abundans*	crude enzyme	60 °C, pH 3.5	2024	[32]
*Thermoascus aurantiacus*	*β*-xylosidase	60 °C, pH 3.5	2023	[42]

**Table 4:** Summary of experimental conditions for the bioconversion of ginsenoside Rc to Rd. This table compares different enzymes or microbial strains and their corresponding reaction parameters.

Source	Enzyme	Conditions	Year	Ref.
*Endophytic Chaetomium globosum*	GH43 Bifunctional Glycosidase	37 °C, pH 7.0	2025	[22]
*Fervidobacterium pennivorans* DSM9078	GH1 *β*-glucosidase	28 °C, pH 7.5	2025	[25]
*Bacillus pumilus*	novel *α*-L-arabinofuranosidase	50 °C, pH 8.0	2025	[45]
*Petroclostridium xylanilyticum*	*β*-glycosidase	60 °C, pH 6.0	2024	[30]
*Bacillus subtilis*	*α*-l-arabinofuranosidase	40 °C, pH 5.5	2024	[46]
*Mucor abundans*	crude enzyme	60 °C, pH 3.5	2024	[32]
*Bacillus subtilis*	*α*-L-arabinofuranosidase	30 °C, pH 7.5	2024	[47]
G9y		45 °C, pH 7.0	2021	[48]

## Data Availability

All new data have been presented in this paper. There are no further data, but the author welcomes questions and discussion.

## Abbreviations

The following abbreviations are used in this manuscript:

Rd	Ginsenoside Rd
AD	Alzheimer’s Disease
PD	Parkinson’s Disease
IS	Ischemic Stroke
